# Editorial: Quantitative Imaging for Clinical Decisions

**DOI:** 10.3389/fonc.2022.858372

**Published:** 2022-03-03

**Authors:** Nandita Maria deSouza, Daniela Elena Oprea-Lager, Laure S. Fournier

**Affiliations:** ^1^ Division of Radiotherapy and Imaging, The Institute of Cancer Research and Royal Marsden NHS Foundation Trust, London, United Kingdom; ^2^ Department of Radiology & Nuclear Medicine, Cancer Centre Amsterdam, Amsterdam University Medical Centers, VU University, Amsterdam, Netherlands; ^3^ Université de Paris, PARCC, INSERM, Radiology Department, AP-HP, Hopital Europeen Georges Pompidou, Paris, France

**Keywords:** decision support, quantitative, computerized tomography, magnetic resonance imaging, positron emission tomography, ultrasound elastography

Ever since the first captivating X-ray images of Mrs. Roentgen’s left hand, medical imaging has been at the heart of clinical decision-making. Over a century later, the explosion in clinically available digital imaging techniques such as computerized tomography (CT), magnetic resonance imaging (MRI) and positron emission tomography (PET) has meant that more objective analysis of images has become desirable to facilitate clinical decisions. Therefore, the demand for quantitative imaging data is increasingly supplementing or sometimes replacing the subjective evaluation of disease visualised on scans. At the simplest level, image quantitation has involved linear measurements of visualised abnormalities and Response Evaluation Criteria In Solid Tumors (RECIST) have been the standard for assessing tumors, their regression, progression and control with treatment. This simple measurement remains at the heart of clinical decision-making in oncology and its role is showcased in a flagship expert statement by Fournier et al “RECIST 20 years on” which discusses the principles underlying RECIST measurements, their reproducibility, limitations and clinical relevance after two decades of use.

The nature of bone lesions has dictated that RECIST measurements are not applicable to the skeleton, so that bony lesions have traditionally been considered non measurable and relied on scoring indices (1). The review by Oprea-Lager et al. challenges this view and describes the newer imaging modalities such as whole-body magnetic resonance imaging (WB-MRI) with diffusion-weighted imaging and positron-emission tomography (PET) including the use of new targeted tracers that open the door to quantifying skeletal pathology. Exploitation of these techniques in order to introduce quantitative imaging for skeletal metastases has been endorsed and advocated by consortia and trial groups (2).

Although ultrasound (US), one of the earliest imaging modalities to be used for clinical decision-making, is not considered directly quantitative, its evaluation of tissue stiffness (a vital clinical sign exploited by clinical palpation) is quantifiable using shear-wave elastography. US shear-wave elastography is emerging as a viable technique (3), particularly in assessing and delineating liver fibrosis and prostate cancer. Hardware and software advances promise that it will be implemented more routinely in clinical practice. The research article by Wei et al investigates its utility as a biomarker for predicting change in biopsy-assigned Gleason score at radical prostatectomy, showing that tissue stiffness can predict upgrading of Gleason score. In future, if performed as part of lesion evaluation prior to US-guided biopsy, this technique has the potential to alter selection of surgical vs. non-surgical management options. Additionally, its use in guiding the biopsy procedure itself or directing other therapeutic strategies such as US-guided high intensity focused ultrasound (HIFU) may become invaluable.

Tissue characterisation has largely been the domain of MRI with biologically-driven multiparameter evaluation dominating the landscape. In neuroimaging, the literature has been dominated by diffusion-weighted imaging. Li et al. examine the non-Gaussian diffusion in glioblastoma multiforme using diffusion kurtosis imaging to investigate whether any of the derived parameters are significant predictors of overall survival. They show that in the multivariate Cox model, the mean kurtosis in the gadolinium-enhanced gross tumor volume pre-radiotherapy was still a significant predictor of overall survival after adjusting effects of age, tumor tissue methylation status and extent of resection. Tissue characterization with quantifiable image data may also be achieved using dual-energy spectral CT (DESCT) (4). Cao et al. utilise spectral CT of the primary tumor in colorectal cancer to predict lymph node metastases, the detection of which remains a holy grail because size criteria are often poorly predictive of involvement. Their nomogram incorporating clinical and DESCT parameters shows clinical potential in this application, which also has been indicated in other cancer types (5).

More recently, a data-driven approach to biomarker extraction has been advocated using radiomics which probe the shape, first-order statistical and texture features within a region-of-interest. The bibliometric analysis by Ding et al. provides an overview of literature related to radiomics in oncology, highlighting artificial intelligence (AI), segmentation method, and use of radiomics for classification and diagnosis in oncology as hotspots. The reproducibility and statistical method of radiomics research, the relation between genomics and radiomics, and the applications of radiomics in sarcoma and intensity-modulated radiotherapy have been identified as research frontiers in the field. The link between radiomics features and histopathology is explored in *ex-vivo* ovarian cancer tissue using images acquired at 9.4T by Tardieu et al. and illustrates the correlation between radiomics features and stromal proportion. A relationship between tissue compartments has been shown in other studies (6), but the association between these features and stromal proportion on histology potentially offers avenues for understanding the biology of this disease by uncovering the histological changes that occur within individual lesions during tumour regression and progression.

Treatment response is an area where quantitative biomarkers are actively desired for clinical decision-making. Hellwig et al. address this in their study in head and neck cancer and develop a random forest based model with dynamic contrast-enhanced parameters to predict treatment response to induction chemotherapy. This is taken further using three-dimensional convolutional neural networks (CNN) in lung cancer by Hou et al. using deep transfer learning to stratify patients into subgroups with different response and progression risks. Their work illustrates the potential of CNN to stratify progression status in patients with epidermal growth factor receptor (EGFR) mutations treated with first-line tyrosine kinase inhibitors. Although such small single centre studies provide a handle on quantitative biomarker discovery, one of the weakest links in parameter generation is the reliability and reproducibility of the segmentation method. Conventionally, this is done manually by trained observers, but computer-aided segmentation is increasingly used (7). This potentially improves the reliability of segmentation methods. Li et al. examine the reproducibility of a computer-aided contouring tool in tumor measurements, and its impact on evaluation of tumor response in terms of RECIST 1.1 criteria. Their data highlight the improvements in interobserver variability that can be achieved with computer aided contouring, which is particularly evident when assigning patients to response categories, thus profoundly impacting individual patient management with regard to therapeutic decisions.

Quantitative imaging is now available with a variety of imaging techniques and there is an explosion in the wealth of parameters that can be derived, particularly with the advent of data-driven approaches of feature extraction. It is important that as imagers and clinicians we are not seduced by the ever-increasing amount of data available, but rather that we select appropriately the data that is truly meaningful and able to reliably influence our clinical decisions. This demands rigor in deriving, qualifying and validating quantitative biomarkers to advance patient management.

## Author Contributions

All authors contributed to the article and approved the submitted version.

## Conflict of Interest

The authors declare that the research was conducted in the absence of any commercial or financial relationships that could be construed as a potential conflict of interest.

## Publisher’s Note

All claims expressed in this article are solely those of the authors and do not necessarily represent those of their affiliated organizations, or those of the publisher, the editors and the reviewers. Any product that may be evaluated in this article, or claim that may be made by its manufacturer, is not guaranteed or endorsed by the publisher.
